# Genetic diagnosis of facioscapulohumeral muscular dystrophy type 1 using rare-variant linkage analysis and long-read genome sequencing

**DOI:** 10.1016/j.gimo.2024.101817

**Published:** 2024-01-29

**Authors:** Kun Li, Daniel Quiat, Fei She, Yuanwei Liu, Rong He, Alireza Haghighi, Fang Liu, Rui Zhang, Steven Robert DePalma, Ying Yang, Wen Wang, Christine E. Seidman, Ping Zhang, Jonathan G. Seidman

**Affiliations:** 1 Department of Cardiology, Beijing Tsinghua Changgung Hospital, School of Clinical Medicine, Tsinghua University, Beijing, China; 2 Department of Genetics, Harvard Medical School, Boston, MA; 3 Department of Pediatrics, Harvard Medical School, Boston, MA; 4 Department of Cardiology, Boston Children’s Hospital, Boston, MA; 5 Cardiovascular Division, Brigham and Women’s Hospital, Boston, MA; 6 Howard Hughes Medical Institute, Harvard University, Boston, MA

**Keywords:** FSHD, Long-read genome sequencing, Myopathy, Nanopore

## Abstract

Facioscapulohumeral dystrophy type 1 (FSHD1) is a progressive, debilitating skeletal myopathy that requires a multimodal approach for complete molecular characterization of pathogenic genotypes. Here, we report genomic analyses of a family with suspected FSHD1. We first performed short-read genome sequencing, followed by parametric linkage analysis using rare variants to map the disease locus to a single 1.7 Mb interval on chromosome 4q35.2 with a logarithm of the odds score of 3.2. We then used ultra-long-read genome sequencing as a single molecular test to genotype a pathogenic FSHD allele containing a 4qA permissive haplotype and 5 KpnI repeat units at the D4Z4 locus. These results demonstrate that genome-wide rare variant–based linkage analysis is a powerful tool for mapping disease loci in families, and ultra-long-read genome sequencing is capable of genotyping pathogenic FSHD1 alleles.

## Introduction

Facioscapulohumeral dystrophy type 1 (FSHD1, OMIM https://www.omim.org/entry/158900) is an autosomal dominant myodystrophy with progressive and regional skeletal muscle involvement and variable disease penetrance.^1^^,^^2^ The pathogenic genetic mechanism of FSHD1 is epigenetic de-repression of the *DUX4* gene encoded within macrosatellite tandem repeats at the D4Z4 locus in the subtelomeric region of chromosome 4q.^3^ Unaffected individuals carry 11-100 highly similar 3.3-kb KpnI repeat units at the D4Z4 locus, each containing 1 copy of the open reading frame of the gene encoding the transcriptional regulator *DUX4*, which is normally expressed during embryonic development and epigenetically repressed in somatic tissues. Affected individuals carry an allele with 1 to 10 KpnI repeat elements and a permissive 4qA haplotype containing a polyadenylation signal within the adjacent pLAM sequence, which in combination result in aberrant expression of *DUX4* that is toxic to skeletal muscle cells.^1^^,^^4^, ^5^, ^6^, ^7^

Molecular diagnosis of FSHD1 has routinely been made by Southern blotting, which requires a substantial amount of input DNA and yields inconclusive results in up to 23% of cases.^8^^,^^9^ More recently, optical genome mapping and molecular combing have been used to assess D4Z4 repeat length on chromosome 4q.^10^^,^^11^ Direct sequencing of the pathogenic allele using short-read genome sequencing or Sanger sequencing is technically difficult because of low-complexity repeats with high guanine-cytosine content and the existence of a highly homologous subtelomeric region on chromosome 10q26.^12^ Newer long-read sequencing methods, such as Oxford Nanopore Technologies (ONT) sequencing platform, are capable of sequencing single DNA molecules that are kilobases in length, enabling superior sequencing of repetitive regions of the human genome.^13^ Long-read genome sequencing methods may permit detection of pathogenic FSHD1 alleles by sequencing the complete D4Z4 repeat array in a single read, allowing for quantification of KpnI repeats and detection of the permissive 4qA disease haplotype. Furthermore, a long-read genome sequencing approach may allow for the concurrent detection of pathogenic variants in the gene *SMCHD1* that result in the closely related facioscapulohumeral dystrophy type 2 (FSHD2, OMIM 158901) and genotyping at other loci associated with myopathy. Here, we use a combination of short- and long-read genome sequencing in a multiplex kindred to map and diagnose FSHD1, respectively.

## Materials and Methods

### Participants

**Figure 1:**
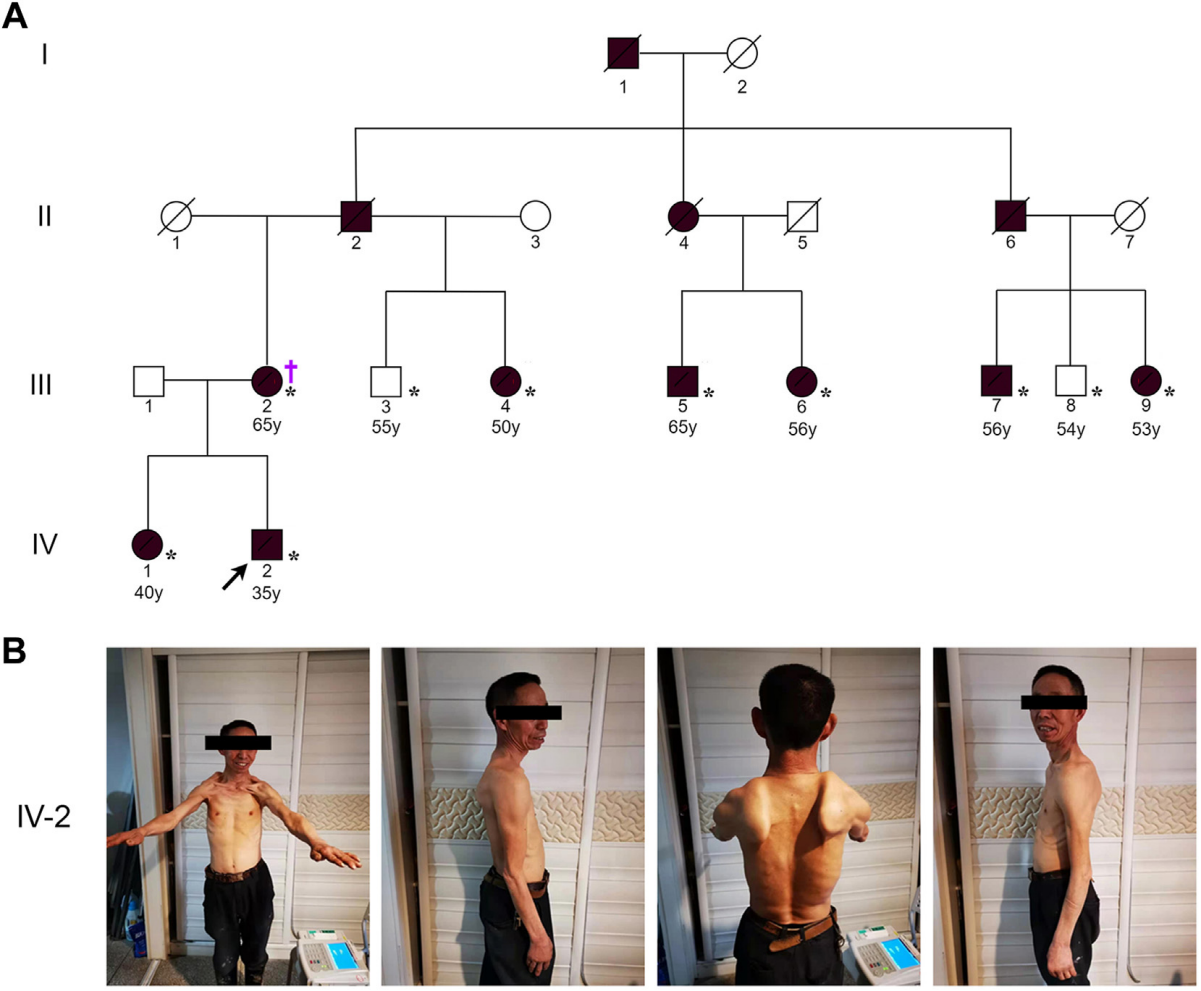
**Pedigree of a kindred with suspected FSHD.** A. A multiplex kindred from rural China with suspected FSHD. The proband (IV-2) is indicated by a black arrow. Asterisks (∗) denote individuals who underwent Illumina short-read genome sequencing, and dagger (†) denotes evaluation by ultra-long nanopore sequencing. Males are represented by squares, females by circles, and affected members by black symbols. Deceased individuals are marked by a diagonal line. B. Phenotype of the proband (IV-2). Physical findings include bilateral atrophy of biceps and pectoralis major, bilateral winged shoulder, and unequal height of shoulders. FSHD, facioscapulohumeral dystrophy.

The cohort analyzed in this manuscript consists of 8 patients and 2 unaffected individuals from a large kindred spanning 4 generations with suspected myopathy recruited at a single center by a single provider. Genomic DNA was extracted from peripheral blood samples of the 8 affected and 2 unaffected family members (Figure 1A).

### Short-read genome sequencing

The NadPrep EZ DNA Library Preparation Module (for Illumina) kit was used for library construction, followed by high-throughput, high-depth sequencing on the Illumina Novaseq platform with 150-bp paired-end reads. Reads underwent adapter trimming, then were aligned to the hg38 reference genome using the Burrows-Wheeler Aligner (BWA-MEM) and processed in accordance with Genome Analysis Toolkit (GATK, Broad Institute) Workflow best practices (Poplin R, Ruano-Rubio V, DePristo M, et al. Scaling accurate genetic variant discovery to tens of thousands of samples. bioRxiv; 2017). Single nucleotide variant (SNV) and indel calls were filtered with the “PASS” filter from Variant Quality Score Recalibration logarithm of the odds scoring, read depth (DP) ≥ 10, and genotype quality ≥ 20, and subsequently phased with Eagle (v2.4.1) using the 1000 Genomes Project Phase 3 reference panel.

### Rare-variant parametric linkage analysis

Under the assumption of an autosomal dominant model based on the pedigree, we filtered variant calls for rare heterozygous biallelic SNVs with a frequency in East Asian subjects in gnomAD (v3) less than 1E-4, then used these haplotype-defining SNV markers to perform a parametric linkage analysis using Merlin (v1.1.2) with the following parameters: dominant model, an estimated population allele frequency of 1E-4, and penetrance of 90%.

### Nanopore sequencing and analysis

High-molecular-weight genomic DNA was prepared by the CTAB method and followed by purification with QIAGEN Genomic kit (Cat# 13343, QIAGEN) for regular sequencing. In addition, 8 to 10 μg of gDNA was size selected (>50 kb) with SageHLS HMW library system (Sage Science) and processed using the Ligation sequencing 1D kit (SQK-LSK109, ONT). Furthermore, 800 ng of DNA library was sequenced on a PromethION device (ONT) at the Genome Center of Grandomics (Wuhan, China). Base calling was performed with Guppy. Quality control metrics were calculated using pycoQC (https://github.com/a-slide/pycoQC). Reads were aligned to the chm13 (v2) reference genome^14^ with minimap2^15^ because of improved representation of repetitive genomic sequence in the chm13 reference sequence. Reads that aligned to chromosome 4q and spanned the D4Z4 repeat array were retained for further analysis. We then used nucmer tool from the Mummer package to perform pairwise sequence alignment with the 3.3 kb KpnI repeat element from GenBank sequence AF117653. Dotplots were generated with Dot (https://github.com/dnanexus/dot).

## Results

### Clinical phenotyping of the affected kindred

We identified a 4 generation kindred with hereditary myopathy from rural China (Figure 1A). The proband was a 32-year-old man who initially presented to care with recurrent chest pain and persistently elevated markers of muscle injury; on further clinical evaluation, he was noted to have muscular atrophy in the shoulder, back, proximal extremities, and scapular winging (Figure 1B). Detailed family history revealed multiple family members with a similar phenotype of symmetrical or asymmetrical, progressive, proximal muscle atrophy, and weakness that demonstrated autosomal dominant mode of inheritance (Figure 1A and Supplemental Table 1). A clinical diagnosis of familial myopathy was made without definitive diagnosis.

### Rare-variant linkage mapping of the disease locus

**Figure 2:**
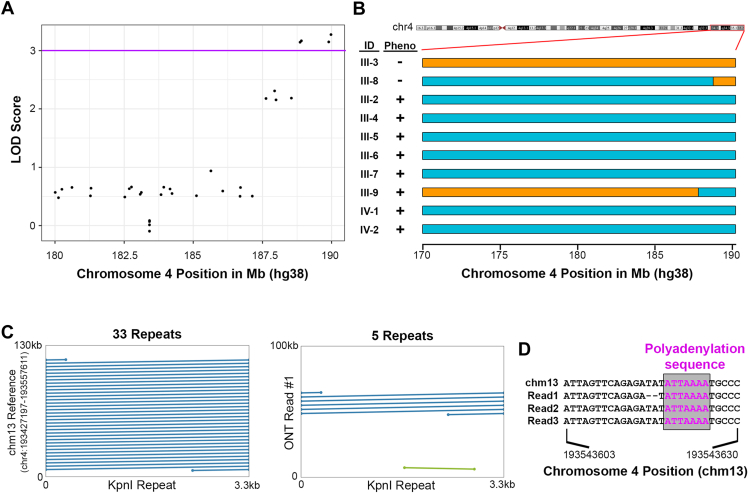
**Genetic mapping and ultra-long read sequencing of the pathogenic allele.** A. Jitter plot of the logarithm of the odds (LOD) score for genetic linkage between rare biallelic SNVs with a population allele frequency of less than 1 × 10^−4^ in East Asian individuals in the gnomAD database indicates linkage at the terminus of chromosome 4q35.2. Genomic coordinates are in hg38. B. Schematic of the haplotypes marked by shared rare variants in affected (Pheno “+”) and unaffected (Pheno “–”) individuals demonstrates the crossing over events that demarcate the pathogenic haplotype. Disease-associated haplotype is depicted in cyan, and other haplotypes are in orange. *X*-axis coordinates are in hg38. C. Sequence alignment of the 3.3 kb KpnI repeat sequence with sequence from 4q in the chm13 reference genome demonstrates the presence of 33 repeats in D4Z4 region of the reference sequence (33 full length dark blue lines). Alignment of ultra-long Oxford Nanopore reads demonstrates the presence a pathogenic allele (“Read #1”) with 5 KpnI repeats in an affected individual. D. Multiple sequence alignment of the chm13 reference genome and sequencing reads containing 5 KpnI repeats (Reads #1-3) demonstrates the presence of the polyadenylation sequence of the 4qA permissive haplotype.

To identify the cause of the familial myopathy, we first performed genome sequencing on 8 affected and 2 unaffected individuals (Figure 1A, asterisk) and did not identify any rare damaging SNVs, indels, genomic structural variants, or splice site variants affecting established or candidate myopathy genes shared across affected individuals. We next sought to leverage genome sequencing data to map the candidate disease locus, specifically the ability to genotype rare SNVs that could mark the haplotype containing the disease allele. Based on the autosomal dominant mode of inheritance, we hypothesized that the haplotype containing the disease allele could be mapped by identifying ultra-rare heterozygous variants shared by all affected individuals and absent from unaffected relatives. We identified 15,417 genome-wide ultra-rare heterozygous biallelic SNVs with an allele frequency of less than 1 × 10^−4^ in East Asian subjects in the genome aggregation database (gnomAD), then performed parametric linkage analysis with these markers and parameters of an autosomal dominant model and estimated penetrance of 90%. Only a single haplotype on the terminus of chromosome 4q35.2 marked by 4 rare biallelic SNVs (Supplemental Table 2) was shared by all affected family members and absent from unaffected individuals, and had a logarithm of the odds score of 3.2 suggesting evidence of genetic linkage (Figure 2A). Evaluating the haplotypes more broadly, we noted 2 crossover events that facilitated mapping of the locus (Figure 2B).

### Molecular diagnosis of FSHD1 by ultra-long-read genome sequencing

The region on chromosome 4q35.2 identified by linkage analysis has previously been associated with FSHD1, a diagnosis that was concordant with the phenotype of the affected individuals in the kindred.^16^ The most distal SNV identified by linkage analysis (4-189975788-A-G (hg38)) is located 87 kilobases (kb) from the centromeric end of the D4Z4 repeat. Clinical genotyping for FSHD1 pathogenic alleles requires analysis of 4q35.2 D4Z4 repeat array length and haplotyping by Southern blot or other techniques.^17^ Given the complex, multimodal protocol for genotyping pathogenic FSHD1 alleles and previous reports of Oxford Nanopore sequencing to assess D4Z4 repeat array length in bacterial artificial chromosome clones,^12^ we hypothesized that third-generation ultra-long-read genome sequencing could be used to define the FSHD1 allele in this kindred and confirm the diagnosis of FSHD1. We performed ultra-long-read Oxford Nanopore genome sequencing in affected individual III-2 (Figure 1A) with a mean read length of 34,910 bp and read length N50 of 62,623 bp (Supplemental Figure 1A and B). We then aligned reads to the chm13 reference genome, which, unlike the hg38 reference sequence, contains a gapless assembly of complex repetitive sequence on 4q35.2. The chm13 reference sequence contains 33 KpnI repeat elements in the D4Z4 array on chromosome 4q35.2 (Figure 2C). We identified 4 sequencing reads from affected individual III-2 that aligned to 4q35.2 and spanned the D4Z4 repeat array. Three reads (66, 100, and 160 kb in length) contained 5 KpnI repeat elements (“Reads #1-3,” Figure 2C, Supplemental Figures 2 and 3) and the 4qA permissive haplotype with ATTAAA polyadenylation sequence (Figure 2D) consistent with a pathogenic FSHD1 haplotype in an affected individual. One 110 kb read contained 11 KpnI repeats (“Read #4,” Supplemental Figure 2B) and did not contain the 4qA polyadenylations sequence, both inconsistent with a pathogenic FSHD1 allele.

## Discussion

We report the use of both short- and long-read genome sequencing to map a familial disease haplotype to the FSHD1 locus and genotype a pathogenic FSHD1 allele present in the disease-associated haplotype. Pathogenic FSHD1 alleles contain a contraction of the D4Z4 macrosatellite repeat array on chromosome 4q, precluding diagnosis by standard sequencing-based diagnostics that poorly assess repetitive genomic regions. Third-generation long-read sequencing enables sequencing of large genomic repeats. This, coupled with the recent release of a telomere-to-telomere reference genome that more accurately incorporates complex repetitive regions, enables the use of reference-based sequencing methods for genotyping of complex repeat alleles. In our study, we obtained 3 sequencing reads from a pathogenic allele among 756,190 total reads that passed quality control, despite achieving ultra-long read lengths with N50 of 62,623 bp. We did not obtain any high-confidence reads fully spanning the nonpathogenic allele. A single read containing 11 KpnI repeats and without the 4qA polyadenylation sequence aligned to the 4q D4Z4 locus, and this read could represent the nonpathogenic allele, mosaicism/rearrangement, or misalignment of a read from the 10q subtelomeric region (Supplemental Figure 4). Increasing the overall sequencing depth, use of adaptive sampling to selectively sequence DNA molecules from areas of interest (4q35.2, *SMCHD1* locus, etc), or performing target capture before sequencing will likely improve the yield of ultra-long reads available for robust molecular analysis. Recently, Hiramuki et al^18^ reported the use of CRISPR/Cas9-targeted Nanopore sequencing to simultaneously identify contracted and hypomethylated 4q-D4Z4 repeats in patients with FSHD1. Advantages of this approach include increased depth of coverage of the 4q-D4Z4 region but notably required a molecular and bioinformatic approach specifically tailored for assessment of the 4q-D4Z4 locus. In contrast, the long-read sequencing method in this analysis used gene/locus agnostic sequencing and alignment, followed by specific assessment of the 4q-D4Z4 locus. Advantages of sequencing-based genotyping include the potential for FSHD1 genotyping in a single assay that can assess genomic structural changes (D4Z4 KpnI repeat number), 4qA/B haplotype status, and damaging variants in *SMCHD1* that are associated with FSHD2. Furthermore, long-read genome sequencing allows simultaneous evaluation of loci for other repeat-mediated myopathies, such as myotonic dystrophy type 1 and 2. We anticipate that continued advances in long-read sequencing technologies, lower sequencing cost, and improved bioinformatic tools for resolving variation in complex genomic regions will improve the ability to evaluate 4q-D4Z4 genotypes using genome-wide approaches.

In addition to the use of long-read sequencing to genotype the pathogenic allele, we used rare variants with a population allele frequency of less than 1 × 10^–4^ for linkage analysis and 4 SNVs were identified with robust evidence of linkage. These markers could be utilized for predictive testing of unaffected family members, albeit recognizing limitations because of potential for recombination events. A limitation of our analysis is that we were only able to genotype the pathogenic allele within the 4q35.2 linkage interval by long-read genome sequencing in a single affected family member and are unable to exclude additional recombination events at the D4Z4 locus in other affected family members, including the proband. Furthermore, clinical genotyping in affected individuals by Southern blotting was unavailable, limiting the completeness of molecular analysis by standard methods.

In summary, our genome sequencing study of a large kindred with FSHD1 demonstrates the use of genome-wide rare variant–based linkage analyses as a powerful tool for the mapping of pathogenic regions in multiplex kindreds and the potential for ultra-long nanopore sequencing as a genetic testing modality for diagnosis of FSHD1.

## Data Availability

All data and code are available on request from the corresponding authors. The raw sequence data reported in this paper have been deposited in the Genome Sequence Archive in National Genomics Data Center China National Center for Bioinformatics/Beijing Institute of Genomics, Chinese Academy of Sciences (GSA-Human: HRA003506) that are publicly accessible at https://ngdc.cncb.ac.cn/gsa-human.

## Conflict of Interest

The authors declare no conflicts of interest.
