# Supplementary material for: Genetic diagnosis of facioscapulohumeral muscular dystrophy type 1 using rare-variant linkage analysis and long-read genome sequencing
Source: Genet Med Open. 2024 Jan 29;2:101817. doi: 10.1016/j.gimo.2024.101817 (PMC11613926; doi:10.1016/j.gimo.2024.101817)
Supplement: Supplemental Material [file mmc1.pdf]

Supplemental Material

Genetic diagnosis of Facioscapulohumeral Muscular Dystrophy Type 1 Using Rare Variant Linkage Analysis and Long Read Genome Sequencing

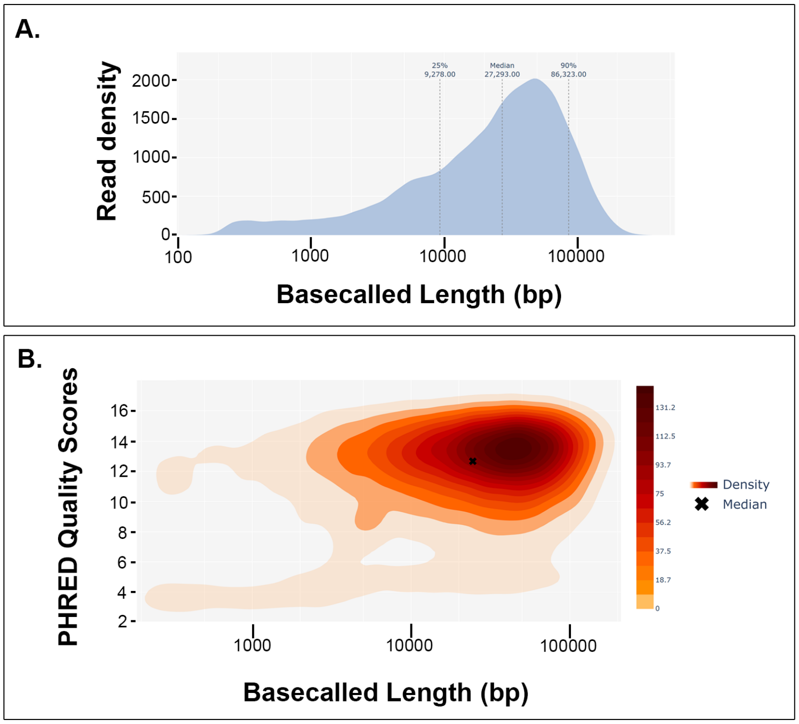

Supplemental Figure 1. Sequencing metrics from Oxford Nanopore Sequencing. (**A**) Read length distribution of all reads that pass quality metrics. Median, 90th percentile, and 25th percentile read lengths are noted. (**B**) Density plot of the relationship between sequencing read length and read quality score for all sequencing reads passing and failing quality metrics.

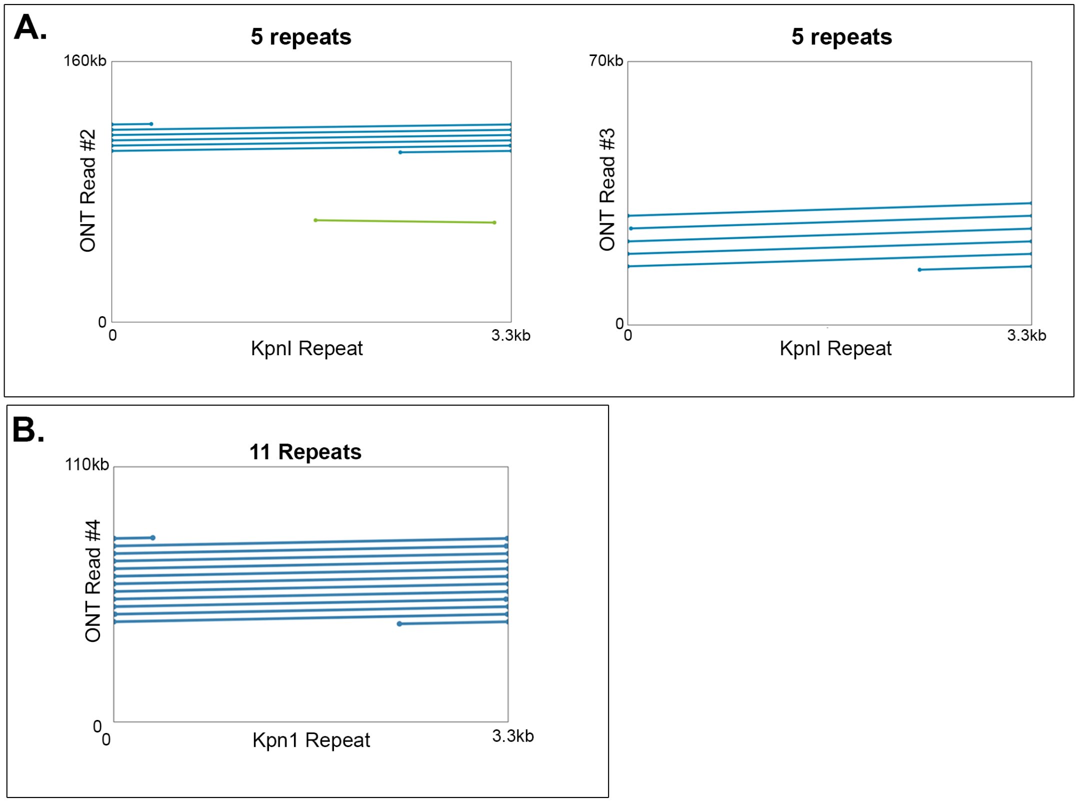

Supplemental Figure 2. Additional nanopore reads aligned to the 4q D4Z4 locus. (A) Sequence alignment of two additional nanopore sequencing reads ('ONT Read #2' and 'ONT Read #3') with the 3.3kb KpnI repeat sequence at D4Z4 locus demonstrate the presence of 5 KpnI repeats, consistent with a pathogenic D4Z4 contraction. (**B**) Sequence alignment of 'ONT Read #4' contains 11 KpnI repeats, inconsistent with a pathogenic repeat contraction.

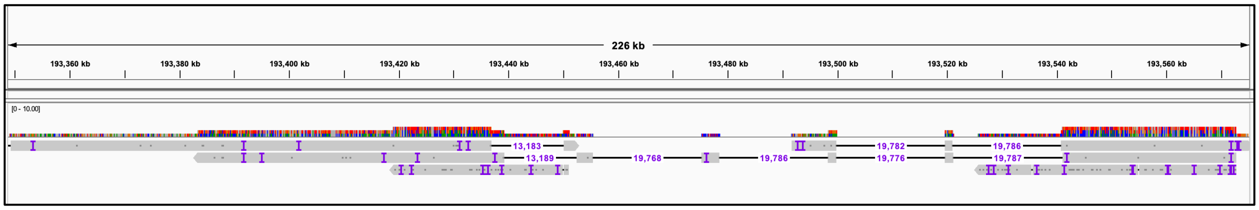

Supplemental Figure 3. Alignment of sequencing reads containing the pathogenic FSHD1 allele to the reference sequence. Integrated Genomics Viewer (IGV) screenshot of 3 Oxford Nanopore reads aligned to the chm13 (v2) reference sequence demonstrates large gaps in read alignment due to D4Z4 repeat contraction.

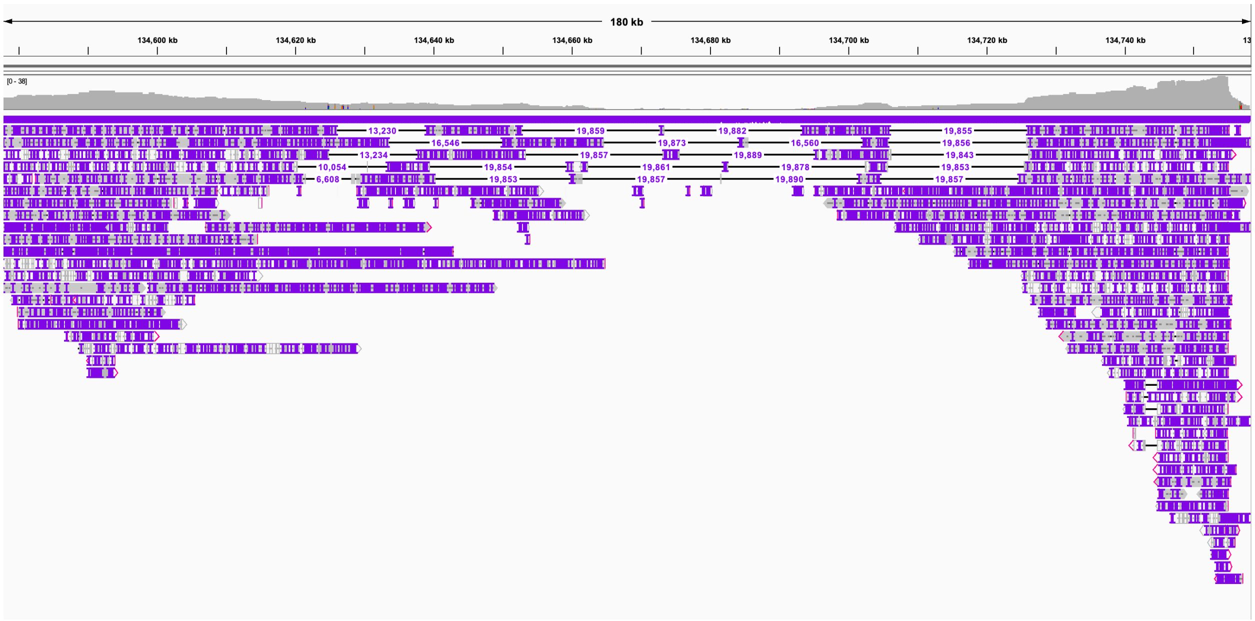

Supplemental Figure 4. ONT Reads aligned to the subtelomeric region of 10q. Visualization of sequencing aligned reads in the subtelomeric region of chromosome 10q (chm13 reference) demonstrates 3 reads with high mapping quality (MQ) and 2 reads with low MQ spanning the D4Z4 repeat array on 10q.

Supplemental Table 1. Clinical manifestations of FSHD in the kindred

	Participant ID							
	III-2	III-4	III-5	III-6	III-7	III-9	IV-1	IV-2
Sex	F	F	M	F	M	F	F	M
Age at clinical evaluation (years)	65	50	65	56	56	53	40	35
Age at Symptom Onset (years)	50	50	14	25	30	23	27	22
**Clinical features of FSHD**								
Weakness affecting the facial or shoulder girdle musculature	+	+	+	+	+	+	+	+
Positive family history	+	+	+	+	+	+	+	+
Asymmetric muscle involvement	+	+	-	+	-	+	+	-
Abdominal weakness	-	-	+	-	+	-	-	-
Retinal vasculopathy or hearing loss in early-onset FSHD	-	-	-	-	-	-	-	-
**Support diagnosis other than FSHD**								
Ptosis or extraocular muscle involvement	-	-	-	-	-	-	-	-
Lingual involvement or difficulty swallowing	-	-	-	-	-	-	-	-
Prominent contractures	-	-	-	-	-	-	-	-
Cardiomyopathy	-	-	-	-	-	-	-	-
Features on EMG or muscle biopsy suggesting an alternative diagnosis	-	-	-	-	-	-	-	-

Supplemental Table 2. Rare Variants in Linkage with the Disease Phenotype

Chromosome	RSID	Position (hg38)	Reference Allele	Alternate Allele	Zygosity
chr4	rs367635682	188855116	T	C	Heterozygous
chr4	rs138969910	188903131	T	A	Heterozygous
chr4	NA	189894661	C	T	Heterozygous
chr4	rs910574891	189975788	A	G	Heterozygous
